# The impact of screening on the survival of colorectal cancer in Shanghai, China: a population based study

**DOI:** 10.1186/s12889-019-7318-8

**Published:** 2019-07-29

**Authors:** Xiaopan Li, Yi Zhou, Zheng Luo, Yi’an Gu, Yichen Chen, Chen Yang, Jing Wang, Shaotan Xiao, Qiao Sun, Mengcen Qian, Genming Zhao

**Affiliations:** 1 School of Public Health, Fudan University, 130 Dong’an Rd, Shanghai, 200032 China; 2 Center for Disease Control and Prevention, Pudong New Area, Shanghai, 200136 China; 3 Fudan University Pudong Institute of Preventive Medicine, Pudong New Area, Shanghai, 200136 China; 4 Shanghai University of Medicine & Health Sciences Affiliated Zhoupu Hospital, Pudong New Area, Shanghai, 201318 China; 5 Department of epidemiology, Columbia University, New York, NY USA; 6 The Key Laboratory of Public Health and Safety of Education Ministry, Fudan University, 138 Yixueyuan Rd, Shanghai, 200032 China

**Keywords:** Colorectal cancer, Screening, Compliance, Survival

## Abstract

**Background:**

Shanghai is one of the earliest cities in developing countries to introduce an organized colorectal screening program for its residents to fight against the rising disease burden of colorectal cancer (CRC). This study aims to investigate the impact of the Shanghai screening program implemented in 2013 on the survival rates of CRC patients.

**Methods:**

We calculated up to 5-year survival rates for 18,592 CRC patients from a representative district of Shanghai during 2002–2016, using data from the Shanghai Cancer Registry. We performed joinpoint regressions to examine temporal changes in the trends of the CRC survival rates. We then conducted Kaplan-Meier and Cox proportional hazards modelling to study the association of the survival rates with screening behaviors of the patients. In all the model specifications, we took into account the gender, age and TNM stage at diagnosis, and level of treatment hospital of the patients.

**Results:**

We find that the annual percentage changes of the survival rates increased faster after somewhere around 2013, however, the differential trends were not significant. Results from the Cox multivariate regression analysis suggest that patients who did not participate in the screening program showed significantly lower cancer-specific survival (hazard ratio (HR) = 1.46; 95% confidence interval (CI): 1.12–1.91) and all-causes survival (HR = 1.37; 95% CI: 1.05–1.77), compared to those who did. Among program participants, delayed colonoscopy was associated with poor cancer-specific survival (hazard ratio (HR) = 2.93; 95% confidence interval (CI): 1.64–5.23) and all-causes survival (HR = 3.29; 95% CI: 1.85–5.84).

**Conclusion:**

Screening participation and high level of colonoscopy compliance can improve the survival of CRC participants.

**Electronic supplementary material:**

The online version of this article (10.1186/s12889-019-7318-8) contains supplementary material, which is available to authorized users.

## Background

Colorectal cancer (CRC) is the third most common types of tumor among males and the second among females around the world [1]. In China, CRC has been the top five most common cancers among both men and women [2]. In recent years, the CRC incidence rate has increased rapidly from a historical low level in several Eastern Asian countries, especially China, which is widely believed to be associated with the adoption of a westernized diet and higher prevalence of obesity and physical inactivity [3, 4].

In developed countries, CRC screening which usually involves a primary screening and a diagnostic colonoscopy, has been shown to be helpful in improving CRC survival for participants, and is responsible for the downward trends of CRC mortality. Thus, CRC screening has been recommended in clinical practice guidelines in these countries [5–7]. However, the benefits of similar screening programs in developing countries are less understood. Due to differences in socioeconomic environments, knowledge and attitudes towards screening, and levels of program compliance, results from developed countries may not well represent the situation in developing countries, where screening programs have just been initiated. Therefore, it is important to study whether the CRC screening program also lead to improved survival for residents in developing societies [8–12].

Shanghai is one of the earliest cities in developing countries to introduce a large community-based colorectal cancer screening program (C-CRCSP) for its residents, which provides a good platform for us to study this issue. The program was launched by the Shanghai government in 2013 and residents aged 50–74 years were set as the target population. In the program, primary care physicians sent screening invitations to the eligible target population. However, the service provided was not exclusively for them if individuals out of the target age range required to take the screening, because the government designed the program as a basic social welfare benefit. Up to the end of 2016, two rounds of the screening have been completed [13, 14].

In this study, we aimed to investigate the impacts of the Shanghai screening program on the survival of CRC patients by examining the trends of up to 5-year CRC survival rates during 2002–2016 and the association of cause-specific and all-causes survival rates with screening behaviors.

## Methods

### Study participants

The study focused on CRC patients diagnosed during 2002–2016 and lived in the Pudong New Area (PNA) of Shanghai, which is the largest district of Shanghai with one-fifth of its total population and a combination of both urban and rural sub-regions [14, 15]. Therefore, the district has been considered good representative of Shanghai [15, 16]. During our study period, there were a total of 18,592 individuals diagnosed among permanent registered residents in the PNA. Among them, 10,904 (58.65%) were diagnosed in their 50–74 years of age, including 66.21% (7,220/10,904) diagnosed before the implementation of the screening program (2002–2012) and 33.79% (3,684/10,904) diagnosed afterwards (2013–2016). Among those diagnosed after the initiation of the program, 23.53% (2817/3,684) of them did not participate in the screening.

According to the data from the reporting system of the screening program, among the 921 individuals who took the primary screening, 403 cases of CRC were reported as a result of the program. We considered these patients being compliant to the program policy since they should have taken a follow-up diagnostic colonoscopy so as to be diagnosed with CRC and reported by both the program and the cancer registry. In contrast, the other 518 participants who were reported with CRC only in the cancer registry rather than also by the program, were thus considered incompliant to the program policy.

### Data collection

We obtained information about demographic characteristics and levels of treatment hospitals for the studied patients from the Shanghai Cancer Registry as of Dec 31, 2017. From the data reporting system of the CRC screening program, we acquired screening behaviors of the patients, such as, participation and program compliance status. From the Shanghai death records, we obtained the death dates, and thus observed the survival status for the patients by the end of 2017 [13, 14, 17].

### Statistical analysis

We used life tables to calculate up to 5-year (1-, 2-, 3-, 4-, and 5-year) survival rates. To explore possible changes in the temporal trends of these survival rates, we performed joinpoint analysis with the Joinpoint Regression Program, Version 4.0.4 (US National Cancer Institute, MD). We then conducted survival analysis using the Kaplan–Meier method with the log-rank test to examine the equality of the survival curves of different subgroups. We performed Cox proportional hazards models to investigate the association of all-causes survival and CRC-specific survival with screening behaviors. To control for the impacts of confounders, we also included the age, TNM stage, gender, level of the treatment hospital of the patients. All statistical tests were two-sided and the analyses were performed using the Statistical Package for the Social Sciences software version 16.0 (SPSS, Inc., Chicago, IL). Statistical significance was set at *P* < 0.05.

### Ethics consideration

Our study did not involve any health-related human participants’ interventions. The data collecting protocols of the cancer registry and the screening program conformed to the ethical guidelines of the 1975 Declaration of Helsinki and were approved by the institutional review board of Centers for Disease Control and Prevention (CDC) of PNA. All patients in this study provided informed consent by themselves or their families. The patients were followed up by primary care physicians at a frequency of no less than 6 months since their diagnosis until death.

## Results

### Sample characteristics

Table 1 presents the number of cases and median survival time for all-causes and cancer-specific deaths by subgroups among CRC patients in Shanghai PNA during 2002–2016. The characteristics of the studied CRC patients by subgroups are shown in Tables 2 and 3. *P*-values of two-sided Chi-square tests on the equality of the distribution rates of these characteristics across subgroups were reported. We find that CRC patients diagnosed after the introduction of the Shanghai screening program were more likely to be male, living in rural sub-regions, treated in secondary hospitals, and in earlier TNM stages at diagnosis, compared to those diagnosed before 2013. The findings still held when the analysis was restricted to patients aged 50–74 years at diagnosis. CRC patients who participated in the primary screening, compared to those who did not, were more likely to be male, living in rural sub-regions, and above 65 years of age and in earlier TNM stages at diagnosis. Conditional on the program participation, we also find that CRC patients who were compliant to the screening policy were more likely to be above 65 years of age and in earlier stages at diagnosis, and treated in secondary hospitals.

**Table 1:** Number of death cases and median survival time by subgroups for colorectal cancer patients in the Shanghai Pudong New Area during 2002–2016

	Total CRC patients		All-causes death		Cancer-specific death	
	n/mean	%/range	n/mean	%/range	n/mean	%/range
Panel A: Number of cases (n, %)						
Diagnosed before the program introduction	12,537	(100.00)	7,554	(60.25)	6,989	(55.75)
Diagnosed after the program introduction	6,055	(100.00)	1,747	(28.85)	1,708	(28.21)
Diagnosed before the program introduction, 50–74 yrs	7,220	(100.00)	3,734	(51.72)	3,475	(48.13)
Diagnosed after the program introduction, 50–74 yrs	3,684	(100.00)	729	(19.79)	723	(19.63)
Participated in the screening program	921	(100.00)	109	(11.83)	103	(11.18)
Never participated in the screening program	2,817	(100.00)	625	(22.19)	619	(21.97)
Compliant to the screening policy	403	(100.00)	27	(6.70)	26	(6.45)
Incompliant to the screening policy	518	(100.00)	82	(15.83)	77	(14.86)
Panel B: Median survival time (n, range)						
Diagnosed before the program introduction	50.57	(0.00–185.53)	17.03	(0.00–179.47)	16.80	(0.00–179.47)
Diagnosed after the program introduction	21.22	(0.00–53.60)	6.40	(0.00–47.10)	6.40	(0.00–47.10)
Diagnosed before the program introduction, 50–74 yrs	62.50	(0.00–185.53)	20.33	(0.00–179.47)	20.72	(0.00–179.47)
Diagnosed after the program introduction, 50–74 yrs	23.88	(0.00–53.60)	9.60	(0.00–47.10)	9.60	(0.00–47.10)
Participated in the screening program	25.33	(0.00–51.83)	9.20	(0.00–41.03)	9.67	(0.00–41.03)
Never participated in the screening program	23.47	(0.00–53.60)	9.50	(0.00–47.10)	9.53	(0.00–47.10)
Compliant to the screening policy	39.50	(0.50–51.83)	11.70	(0.50–41.03)	12.60	(0.50–41.03)
Incompliant to the screening policy	17.13	(0.00–50.93)	5.72	(0.00–35.70)	5.73	(0.00–35.70)

**Table 2:** Characteristics of the colorectal patients in the Shanghai Pudong New Area during 2002–2016, by subgroups categorized according to time and age at diagnosis

	Diagnosed *before* program introduction	Diagnosed *after* program introduction	*P value*	Diagnosed *before* program introduction, 50–74 yrs	Diagnosed after program introduction, 50–74 yrs	*P value*
	(n)	(n)		(n)	(n)	
Gender						
Male	6,706	3,492	< 0.001	4,034	2,229	< 0.001
Female	5,831	2,563		3,186	1,455	
Age						
≤ 65	5,010	2,430	> 0.050	3,845	2,061	< 0.050
> 65	7,527	3,625		3,375	1,623	
Sub region						
Rural	7,064	3,638	< 0.001	4,076	2,195	< 0.050
Urban	5,473	2,417		3,144	1,489	
Level of treatment hospital						
Secondary	4,668	2,561	< 0.001	2,462	1,358	< 0.050
Tertiary	7,869	3,494		4,758	2,326	
TNM stage						
I + II	3,317	1,727	< 0.050	2,135	1,161	< 0.050
III + IV	4,149	1,927		2,464	1,183	
Unknown	5,071	2,401		2,621	1,340

**Table 3:** Characteristics of the colorectal patients in the Shanghai Pudong New Area during 2002–2016, by subgroups categorized according to screening behaviors

	Participated the screening program	Not Participated the screening program	*P value*	Compliant to the screening policy	Incompliant to the screening policy	*P value*
	(n)	(n)		(n)	(n)	
Gender						
Male	519	1,744	< 0.050	227	292	> 0.050
Female	402	1,073		176	226	
Age						
≤ 65	374	1,705	< 0.001	190	184	< 0.001
> 65	547	1,112		213	334	
Sub region						
Rural	565	1,617	< 0.050	254	311	> 0.050
Urban	356	1,200		149	207	
Level of treatment hospital						
Secondary	342	1,028	> 0.050	134	208	< 0.050
Tertiary	579	1,789		269	310	
TNM stage						
I + II	412	837	< 0.001	226	186	< 0.001
III + IV	212	962		69	143	
Unknown	297	1,018		108	189	

### Trends of up to 5-year CRC survival rates in the Shanghai PNA

**Fig. 1:**
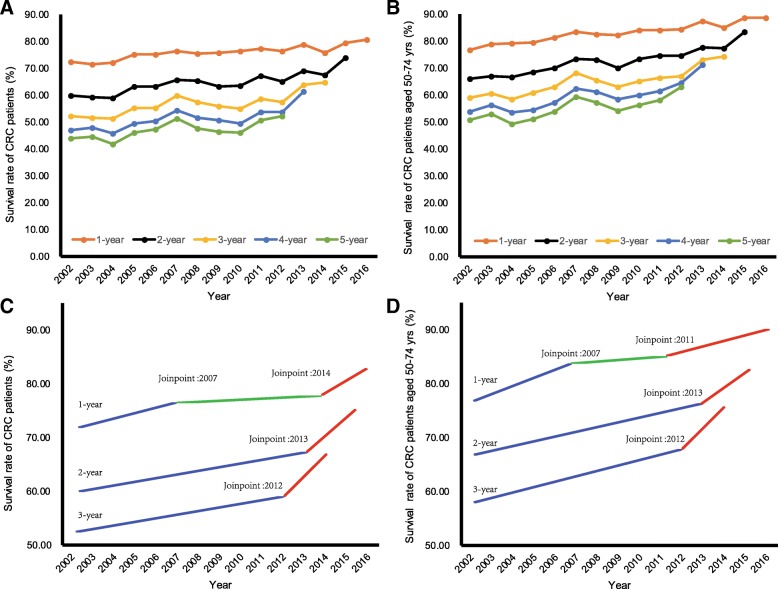
The temporal trends of 1-,2-,3-,4-,5-year survival rates of colorectal cancer patients in the Shanghai PNA during 2002–2016. (**a** and **c** for all Patients; **b** and **d** for patients diagnosed at 50–74 years of age)

Figure 1a plots up to 5-year survival rates for CRC patients in the Shanghai PNA over years. Results for patients aged 50–74 years at diagnosis are reported in Fig. 1b. All survival rates have been increasing since 2002. In 2016, the 1-, 2-, 3-, 4-, and 5-year survival rates were 80.73,74.12,64.74,61.37, and 52.37% for all CRC patients, respectively. And the corresponding results for those aged 50–74 years at diagnosis were 88.64, 83.58, 74.29, 71.18, and 63.10%, respectively (Additional file 1: Table S1).

Results of joinpoint analyses for 1-,2-,3-,4-,and 5-year CRC survival rates suggest similar temporal trends (Table 4). For all CRC patients (Fig. 1c) and those aged 50–74 years (Fig. 1d) at diagnosis, we observed significant (*P* < 0.001) upward trends in all survival rates during 2002–2016. We find that the most recent joinpionts in the temporal trends of the survival rates occurred around the introduction year of the Shanghai screening program: 2014 for 1-year survival rates, 2013 for 2-year survival rates, and 2012 for 3-year survival in the analysis for all CRC patients. Sizes of the upward trends in the above survival rates became larger in periods following the years where the most recent joinpoints occurred, suggesting more rapid increasing of CRC survival rates. However, the differential trends were generally not significant.

**Table 4:** Trends of 1-, 2-, 3-, 4-, and 5-year survival rates for CRC patients in the Shanghai Pudong New Area during 2002–2016, results from joinpoint regression analysis

	Trend 1		Trend 2		Trend 3		Total trend	
Survival rate	Years	APC	Years	APC	Years	APC	Years	APC
All patients								
1-year	2002–2007	1.23*	2007–2014	0.27	2014–2016	2.27	2002–2016	0.70**
2-year	2002–2013	1.09*	2013–2015	3.91			2002–2015	1.32**
3-year	2002–2012	1.20*	2012–2014	5.57			2002–2014	1.60**
4-year	2002–2013	1.78**						
5-year	2002–2012	1.53**						
Aged 50-74 yrs. at diagnosis								
1-year	2002–2007	1.41*	2007–2011	0.39	2011–2016	1.13*	2002–2016	0.92**
2-year	2002–2013	1.32**	2013–2015	3.88			2002–2015	1.53**
3-year	2002–2012	1.37**	2012–2014	5.27			2002–2014	1.73**
4-year	2002–2013	2.05**						
5-year	2002–2012	1.87**						

Notes: Annual percent changes, APC; *, *P* < 0.05; **, *P* < 0.001

### Variations in CRC survival rates across different subgroups

**Fig. 2:**
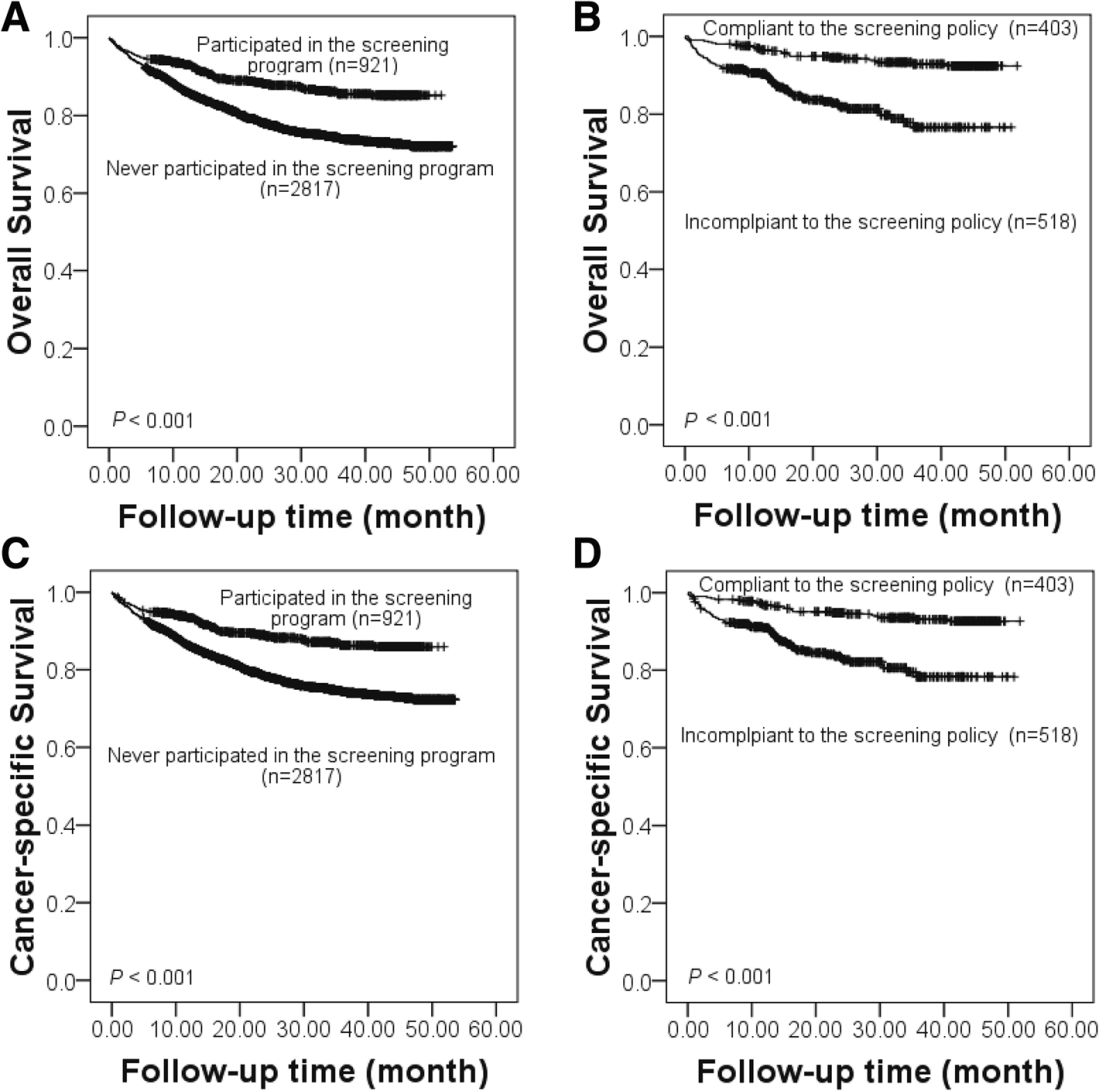
Variations in all-causes and cancer-specific survival of patients with colorectal cancer by screening behaviors. (**a** and **c** for comparisons between participants and non-participants; **b** and **d** for comparisons between compliance group and incompliance group)

Results of the Kaplan-Meier analysis on all-causes survival rate and cancer-specific survival rate for CRC patients who participated and never participated in the Shanghai screening program are shown in Fig. 2a and c. Corresponding results for program participants who were compliant and incompliant to the screening policy are presented in Fig. 2b and d. There were significant differences in both types of survival rate across the subgroups (*P* < 0.001). The all-causes survival rate and the CRC-specific rate of the program participant group were significantly higher than the non-participant group. And among the CRC patients who participated the program, both survival rates of the policy compliance group were significantly higher than those of the policy non-compliance group.

Results of the Cox proportional hazards analysis of cancer-specific and all-causes death in the screening program participants and non-participants group are shown in Panel A of Table 5. Among patients diagnosed after the introduction of the Shanghai CRC screening program, the hazard ratio of the non-participants group was significantly higher (1.46, 95% CI:1.12–1.91), compared with the risk of cancer-specific death for CRC patients who participated in the screening program. Results of the control variables suggest that there were significant differences in the survival rates associated with the gender, level of the treatment hospital, and age and TNM stage at diagnosis of the patients.

**Table 5:** Cox proportional hazards analysis of cancer-specific death and all-causes death for subgroups of CRC patients

	Cancer-specific death			All-causes death		
	HR	(95% CI)	*P value*	HR	(95% CI)	*P value*
Panel A: For CRC patients diagnosed after the introduction of the screening program						
Program participation (Ref. group: Participants of the program)						
Non-participants of the program	1.46	(1.12–1.91)	0.006	1.37	(1.05–1.77)	0.019
Gender (Ref. group: Female)						
Male	1.24	(1.06–1.45)	0.008	1.23	(1.05–1.44)	0.01
Age at diagnosis (Ref. group: ≤65 yrs)						
> 65 yrs	1.49	(1.28–1.74)	< 0.001	1.49	(1.28–1.73)	< 0.001
Sub-region (Ref. group: Rural)						
Urban	0.96	(0.82–1.12)	0.589	0.95	(0.81–1.10)	0.475
Level of treatment hospital (Ref. group: Tertiary)						
Secondary	1.5	(1.12–1.91)	< 0.001	1.49	(1.28–1.73)	< 0.001
TNM Stage (Ref. group: I + II)						
III + IV	1.75	(1.58–1.94)	< 0.001	1.77	(1.60–1.95)	< 0.001
Panel B: For CRC patients who participated in the screening program						
Screening policy compliance (Ref. group: Compliant to the screening policy)						
Incompliant to the screening policy	2.93	(1.64–5.23)	< 0.001	3.29	(1.85–5.84)	< 0.001
Gender (Ref. group: Female)						
Male	1.91	(1.13–3.22)	0.015	1.73	(1.06–2.84)	0.029
Age at diagnosis (Ref. group: ≤65 yrs)						
> 65 yrs	1.11	(0.67–1.85)	0.674	1.18	(0.72–1.93)	0.506
Sub-region (Ref. group: Rural)						
Urban	0.91	(0.53–1.56)	0.718	0.86	(0.51–1.46)	0.579
Level of treatment hospital (Ref. group: Tertiary)						
Secondary	1.31	(0.80–2.14)	0.283	1.34	(0.84–2.15)	0.224
TNM Stage (Ref. group: I + II)						
III + IV	8.55	(4.54–16.13)	< 0.001	7.55	(4.17–13.55)	< 0.001

Notes: Colorectal cancer, CRC; Hazard ratio, HR.

Panel B of Table 5 presents Cox regression results for cancer-specific death and all-causes death in the policy compliance group and the policy incompliance group. Among CRC patients who participated in the screening program, the hazard ratio of those incompliant to the screening policy was significantly higher (2.93, 95% CI: 1.64–5.23), compared to the risks of all-causes death for those compliant to the policy. We also find that the gender and TNM stage at diagnosis of the patients were significantly associated with the survival rate. We find no significant differences in the outcome related with the age, sub-region of residence, and level of the treatment hospital. All the above results remained the same for all-causes death.

In order to check if our models satisfy the assumption of the Cox proportional model, we generated time dependent covariates by creating interactions of the control variables and a function of survival time. We repeated the above analysis by including the interaction terms in the regression. We find that none of the time dependent covariates were significant, suggesting that the control variables were proportional. Results are reported in Additional file 2: Table S2 and Additional file 3: Table S3.

## Discussion

The CRC screening program in Shanghai was implemented in 2013. We investigated impacts of this program on survival from different perspectives, by following up CRC patients diagnosed during 2002–2016 and lived in the Shanghai Pudong New Area and.

The survival rates of the CRC patients exhibited significant upward trends. Results of the trend analysis further suggested that the increases in the annual percentage changes of these rates became more rapid around the introduction of the screening program, though the differential trends were not significant, possibly due to limited observation time. The results were similar for the whole sample and for patients within the target age range of the screening program. There are two possible explanations for these findings. First, the program did not exclude individuals out of the target age range if they voluntarily requested to take the screening, because the program was carried out as a welfare benefit for the Shanghai residents. Second, the impact of the screening program may have exerted a spillover effect on the non-target population via health promotion and education provided by the primary care physicians as a part of the program [18].

Results from the Cox regression models suggested that CRC patients who participated in the screening program or well followed the screening policy enjoyed better survival outcomes compared to those who did not. The differences across different subgroups remained sizable and significant when other confounding factors, as suggested by previous evidence [19, 20], were controlled for. The findings suggest that the screening program benefited the at-risk population by increasing their life expectancy [21].

This study contributes to the literature along several dimensions. Previous evidence on the long-term benefits of screening in developing societies is generally based on results from model projections by adapting experiences from developed countries to the characteristics of their own settings [22]. In contrast, this study evaluated the Shanghai CRC screening program within 3 years after its initiation and thus provides real-world evidence.

Typically, a longer follow-up period is needed to draw more robust conclusions on the association between a screening program and disease survival [18, 23]. However, the immediate evaluation presented by this study is important for improving the delivery and performance of the screening program in Shanghai, as well as other similar programs in developing societies, due to the following reasons. First, the governments in developing societies have devoted substantial resources to carry out a large-scale organized CRC screening program. With less sufficient healthcare provision in these areas, the workload of the healthcare providers are generally quite heavy. However, the program further assigned screening tasks to the physicians in addition to their routine work. Therefore, evidence on the benefits of the screening are crucial for the government to consider whether to continue the program. And such evidence may also improve the performance of the program by increasing the incentives of the physicians. Second, the policy compliance rate of the screening program in developing countries are much lower than that in developed countries, because the program is not well accepted by the public. Confirmed benefits of the screening program may help regulate the screening behavior of the participants, thus leading to better performance of the program.

Our study has limitations. First, due to the short observation period after the introduction of the program, we were not able to examine changes in 4-, and 5-year survival rates for CRC patients diagnosed in 2013–2016. Second, though the study confirms the effectiveness of the screening program on CRC survival, it is still not sure if the gains outweighs the costs of carrying out the program for developing countries given the constrained healthcare resources and low colonoscopy compliance rate. Cost-effectiveness analyses of the program will be the focus of future research.

## Conclusions

Consistent with previous studies from developed countries, our results suggest that the Shanghai organized CRC screening program has been associated with improved survival for the CRC patients. CRC patients who participated in the screening program and were compliant to the screening policy enjoyed better survival outcomes, compared to their counterparts.

## Additional files

Additional file 1: **Table S1.** 1-, 2-, 3-, 4-, and 5-year survival rates of CRC patients in the Shanghai PNA during 2002–2016. (DOCX 16 kb)

Additional file 2: **Table S2.** Results of Cox regressions including time-dependent age. (DOCX 15 kb)

Additional file 3: **Table S3.** Results of Cox regressions including time-dependent TNM stage. (DOCX 15 kb)

## Data Availability

The data that support the findings of this study are available from Centers for Disease Control and Prevention of the Pudong New Area, Shanghai but restrictions apply to the availability of these data. The data was used under license for the current study, and so are not publicly available. Data is however available from the authors upon reasonable requests and with permissions of Center for Disease Control and Prevention of the Pudong New Area.

## Abbreviations

APC: Annual percent change
CRC: Colorectal cancer
HR: Hazard ratio
PNA: Pudong New Area
TNM: Tumor Node Metastasis
