# Supplementary material for: The impact of screening on the survival of colorectal cancer in Shanghai, China: a population based study
Source: BMC Public Health. 2019 Jul 29;19:1016. doi: 10.1186/s12889-019-7318-8 (PMC6664771; doi:10.1186/s12889-019-7318-8)
Supplement: Supplementary file 1 — Table S1. 1-, 2-, 3-, 4-, and 5-year survival rates of CRC patients in the Shanghai PNA during 2002–2016. (DOCX 16 kb) [file 12889_2019_7318_MOESM1_ESM.docx]

**Table S1: 1-, 2-, 3-, 4-, and 5-year survival rates of CRC patients in the Shanghai PNA during 2002-2016**

|  |  |  | CRC patients (%) | |  |  | Patients aged 50-74 years at CRC diagnosis (%) | | | | |
| --- | --- | --- | --- | --- | --- | --- | --- | --- | --- | --- | --- |
| Year | 1-year | 2-year | 3-year | 4-year | 5-year |  | 1-year | 2-year | 3-year | 4-year | 5-year |
|  |  |  |  |  |  |  |  |  |  |  |  |
| 2002 | 72.29 | 59.81 | 52.20 | 47.09 | 44.11 |  | 76.71 | 66.06 | 59.04 | 53.82 | 50.80 |
| 2003 | 71.52 | 59.29 | 51.72 | 48.05 | 44.61 |  | 79.01 | 66.93 | 60.59 | 56.44 | 53.07 |
| 2004 | 72.04 | 59.07 | 51.27 | 45.80 | 41.95 |  | 79.30 | 66.73 | 58.60 | 53.60 | 49.17 |
| 2005 | 75.16 | 63.22 | 55.41 | 49.58 | 46.10 |  | 79.48 | 68.39 | 60.79 | 54.56 | 51.22 |
| 2006 | 75.11 | 63.35 | 55.38 | 50.41 | 47.42 |  | 81.48 | 70.02 | 62.95 | 57.14 | 54.00 |
| 2007 | 76.45 | 65.74 | 59.95 | 54.42 | 51.20 |  | 83.62 | 73.42 | 68.16 | 62.44 | 59.35 |
| 2008 | 75.52 | 65.26 | 57.32 | 51.70 | 47.64 |  | 82.65 | 73.18 | 65.45 | 61.37 | 57.14 |
| 2009 | 75.72 | 63.14 | 55.81 | 50.64 | 46.58 |  | 82.30 | 70.08 | 63.06 | 58.57 | 54.21 |
| 2010 | 76.45 | 63.51 | 55.00 | 49.59 | 45.97 |  | 84.21 | 73.41 | 65.32 | 60.05 | 56.41 |
| 2011 | 77.42 | 67.21 | 58.72 | 53.80 | 50.75 |  | 84.11 | 74.78 | 66.33 | 61.54 | 58.26 |
| 2012 | 76.41 | 65.05 | 57.38 | 53.91 | 52.37 |  | 84.41 | 74.56 | 67.08 | 64.59 | 63.10 |
| 2013 | 79.03 | 68.99 | 63.82 | 61.37 |  |  | 87.45 | 77.71 | 73.19 | 71.18 |  |
| 2014 | 75.91 | 67.43 | 64.74 |  |  |  | 85.00 | 77.34 | 74.29 |  |  |
| 2015 | 79.46 | 74.12 |  |  |  |  | 88.71 | 83.58 |  |  |  |
| 2016 | 80.73 |  |  |  |  |  | 88.64 |  |  |  |  |
