# Supplementary material for: The impact of screening on the survival of colorectal cancer in Shanghai, China: a population based study
Source: BMC Public Health. 2019 Jul 29;19:1016. doi: 10.1186/s12889-019-7318-8 (PMC6664771; doi:10.1186/s12889-019-7318-8)
Supplement: Supplementary file 3 — Table S3. Results of Cox regressions including time-dependent TNM stage. (DOCX 15 kb) [file 12889_2019_7318_MOESM3_ESM.docx]

Table S3: Results of Cox regressions including time-dependent TNM stage

|  | Cancer-specific mortality | | |  | All-causes mortality | | |
| --- | --- | --- | --- | --- | --- | --- | --- |
|  |  |  |  |  |  |  |  |
|  | HR | (95% CI) | *P value* |  | HR | (95% CI) | *P value* |
|  |  |  |  |  |  |  |  |
| **Panel A: For CRC patients diagnosed after the introduction of the screening program** | | | | | |  |  |
| Time-Dep TNM | 0.994 | (0.985-1.004) | 0.226 |  | 0.994 | (0.985-1.004) | 0.218 |
| Program participation (Ref. group: Participated in the screening ) |  |  |  |  |  |  |  |
| Never participated | 1.458 | (1.115-1.907) | 0.006 |  | 1.365 | (1.052-1.772) | 0.019 |
| Gender (Ref. group: female) |  |  |  |  |  |  |  |
| Male | 1.236 | (1.055-1.448) | 0.009 |  | 1.227 | (1.049-1.435) | 0.011 |
| Age at diagnosis (Ref. group:≤65 yrs ) |  |  |  |  |  |  |  |
| >65 yrs | 1.494 | (1.284-1.737) | <0.001 |  | 1.491 | (1.283-1.732) | <0.001 |
| Sub-region (Ref. group: Rural) |  |  |  |  |  |  |  |
| Urban | 0.957 | (0.821-1.116) | 0.574 |  | 0.944 | (0.811-1.100) | 0.462 |
| Level of treatment hopspital (Ref. group: Tertiary) |  |  |  |  |  |  |  |
| Secondary | 1.494 | (1.284-1.739) | <0.001 |  | 1.490 | (1.282-1.731) | <0.001 |
| TNM Stage (Ref. group: I+II) |  |  |  |  |  |  |  |
| III+IV | 1.884 | (1.613-2.201) | <0.001 |  | 1.898 | (1.626-2.214) | <0.001 |
|  |  |  |  |  |  |  |  |
| **Panel B: For CRC patients who participated in the screening program** | | | | | |  |  |
| Time-Dep TNM | 1.002 | (0.944-1.065) | 0.939 |  | 0.995 | (0.942-1.051) | 0.851 |
| Screening policy compliance (Ref. group:Compliant to the screening policy) |  |  |  |  |  |  |  |
| Incompliant to the screening policy | 2.929 | (1.640-5.230) | <0.001 |  | 3.292 | (1.852-5.851) | <0.001 |
| Gender (Ref. group: female) |  |  |  |  |  |  |  |
| Male | 1.909 | (1.132-3.220) | 0.015 |  | 1.734 | (1.057-2.844) | 0.029 |
| Age at diagnosis (Ref. group:≤65 yrs ) |  |  |  |  |  |  |  |
| >65 yrs | 1.115 | (0.672-1.848) | 0.674 |  | 1.181 | (0.723-1.930) | 0.507 |
| Sub-region (Ref. group: Rural) |  |  |  |  |  |  |  |
| Urban | 0.905 | (0.526-1.555) | 0.717 |  | 0.863 | (0.510-1.460) | 0.582 |
| Level of treatment hopspital (Ref. group: Tertiary) |  |  |  |  |  |  |  |
| Secondary | 1.308 | (0.801-2.137) | 0.283 |  | 1.342 | (0.836-2.155) | 0.223 |
| TNM Stage (Ref. group: I+II) |  |  |  |  |  |  |  |
| III+IV | 8.310 | (3.148-21.936) | <0.001 |  | 8.059 | (3.241-20.043) | <0.001 |
